# Annotations

**Published:** 1894-01-27

**Authors:** 


### ANNOTATIONS.

### Private—for Doctors only.

A NEW company is being formed, in which the writer has been invited to take shares, for the sale of a well-known medicinal water. A list of directors is published with the prospectus, and the first name on the list is that of a practising medical man. The prospectus is marked "Private.—For distribution to members of the medical profession only." We do not, at present, give the name of the company, or of the medicinal water it proposes to secure the monopoly of, or of the medical man who heads the list of directors. But we do wish to ask the members of our profession to consider well before they embark in an undertaking of this character. No one will suspect any writer in THE HOSPITAL of desiring to unduly limit the freedom of members of the medical profession. But it is nevertheless true that whilst all honest things are lawful for a medical man, all honest things are not expedient. Is it expedient, is it proper, is it right, that a medical man should have a clear money interest in prescribing for every one of his patients a particular remedy, which may sometimes be suitable, sometimes unsuitable, and sometimes dangerous? There is the whole point of the business. We maintain that no doctor should have a money interest in any single article which he is called upon to prescribe. His mind should be absolutely free from bias of all kinds, in order that he may have no inducement whatever to select for his patient one remedy rather than another. We further maintain that no practitioner can be entirely free from bias who has a money interest in the prescribing of one particular remedy. What would our patients think of any of us if they knew that every bottle of mineral water we ordered for them put a fractional profit into our own pockets? Their loyal confidence would be gone, and properly gone. It is our serious conviction that if this company lives and is followed by other similar companies, the degradation of the medical profession will be markedly deepened.

### Undignified Preventive Medicine.

IF Professor C. S. Roy, of Cambridge, be an impartial witness, there have been some rather high-handed proceedings in connection with the appointment of the first "director" of the British Institute of Preventive Medicine. The institute is now so far beyond the embryonic stage that it is beginning to assume what will doubtless prove to be its permanent form. The present is therefore a critical period, and the appointment of the "director" one of the most critical facts of the period. Professor Roy informs the scientific world, in the pages of *Nature*, that a director has been appointed, not according to the established methods which regulate such appointments, namely, by the public advertising for candidates, and the open selection of the fittest, but by the very decided and not-to-be-resisted insistence of two of the committee's officials, Sir Joseph Lister, chairman of the Council, and Sir Henry Roscoe, its treasurer. Professor Roy himself, in consequence of what he considers the extreme unfairness and injudiciousness of the methods employed in the making of the directorial appointment, has felt it necessary to resign his office of honorary secretary. Assuming for the moment the general correctness of Professor Roy's asseverations, we cannot but deeply regret that anything should have been done in connection with the beginnings of so important an institution as the Institute of Preventive Medicine but what was of the fairest, the most open, the most deliberate, and the most judicial character. The beginnings of this new British Institute are of historical significance; and that some of the most important of its early acts should be tainted by even a suspicion of "jobbery" is as undesirable as anything can possibly be. It is our hope that the chairman and treasurer may be able to put another complexion upon Professor Roy's account of the matter.

### Materialism, Madness, and Poor Humanity.

ONE of the reforms most urgently called for in our judicial system is the creation of a "Tribunal of Sanity," as we may well call it until a better name is found. Lawyers, moralists, theologians, and all sorts of persons who wish to do justice to "poor humanity" will do well to carefully study a small pamphlet written by Dr. T. S. Clouston, Medical Superintendent of the Morningside Asylum, Edinburgh. The pamphlet has for its title, "The Mental Symptoms of Myxœdema, and the Effect on Them of Thyroid Feeding." Myxœdema, it may be briefly stated, is a disease of the body, pure and simple, at any rate at the outset. The man or woman who is the victim of the disease swells all over and increases in weight, sometimes by several stones. What we desire lawyers and theologians to consider is, first, the peculiar effect upon the mind and character which this purely material change produces; and secondly, the extraordinary fact, discovered within the past few years, that the feeding of a person thus diseased, with the thyroid glands of animals, not only cures the bodily disease, but restores the mind to perfect and permanent sanity. The following are a few of the mental changes noted in particular individuals: "One lady described in detail conversations that never could have taken place." Think of this lawyers and judges! "She was not able to distinguish between the remembrance of fancies and the recollection of real events." In other words she could tell a lie with a circumstance, and could not help it. One woman "imagined that everything smelt of gunpowder"; another "that she was continually made to breathe gas." In some females "the power of intense emotion was diminished . . . the patients cared less for their husbands and their children" than they had done before; some were "strikingly indifferent"; . . . "of "acts of violence and destruction were seen in two" of the cases. Now let the effect of feeding with thyroid gland be noted by those who insist upon moral responsibility in every person who is not technically mad. The cases of two of the women, noted above, will suffice. Both these women were speedily cured of the mental and moral aberrations by the eating of thyroid gland—mental and moral ills alike cured by material means. What we wish to insist upon is that this condition of irresponsibility neither judges, lawyers, nor jurors are capable of deciding upon. Competent doctors are capable, can indeed see at a glance from the physical condition. Does not justice demand, when such persons come into collision with the law, as they often do, that a competent medical tribunal shall first decide the fact of their responsibility or irresponsibility? We punish and imprison where we should heal and cure. This is unscientific, it is grossly unjust, and it is unworthy of the intelligence of an intelligent nation.

